# Histologic tumor type as a determinant of survival in hormone receptor-positive, HER2-negative, pT1-3 invasive ductal and lobular breast cancer

**DOI:** 10.1186/s13058-023-01745-x

**Published:** 2023-11-22

**Authors:** Menekse Göker, Hannelore Denys, An Hendrix, Olivier De Wever, Koen Van de Vijver, Geert Braems

**Affiliations:** 1 Department of Gynaecology, Ghent University Hospital, Ghent, Belgium; 2 Department of Medical Oncology, Ghent University Hospital, Ghent, Belgium; 3 Laboratory for Experimental Cancer Research, Ghent University Hospital, Ghent, Belgium; 4 Department of Histopathology, Ghent University Hospital, Ghent, Belgium

**Keywords:** Breast cancer, Invasive Lobular cancer, Invasive ductal cancer, Survival

## Abstract

**Purpose:**

The aim of the study was to compare the difference in survival between invasive ductal (IDC) and lobular carcinoma (ILC).

**Methods:**

Data of patients (n = 1843) with a hormone receptor-positive, HER2-negative, pT1-3 IDC or ILC cancer without distant metastasis, treated at the Ghent University Hospital over the time period 2001–2015, were analyzed.

**Results:**

ILC represented 13.9% of the tumors, had a higher percentage of pT3 and pN3 stages than IDC, lymphovascular space invasion (LVSI) was less present and Ki-67 was mostly low. 73.9% of ILCs were grade 2, whereas IDC had more grade 1 and grade 3 tumors. Kaplan–Meier curves and log-rank testing showed a significant worse DFS for ILC with pN ≥ 1 than for their IDC counterpart. In a multivariable Cox regression analysis the histologic tumor type, ductal or lobular, was a determinant of DFS over 120 months (IDC as reference; hazard ratio for ILC 1.77, 95% CI 1.08–2.90) just as the ER Allred score (hazard ratio 0.84, 95% CI 0.78–0.91), LVSI (hazard ratio 1.75, 95% CI 1.12–2.74) and pN3 (hazard ratio 2.29, 95% CI 1.03–5.09). Determinants of OS over ten years were age (hazard ratio 1.05, 95% CI 1.02–1.07), LVSI (hazard ratio 3.62, 95% CI 1.92–6.82) and the ER Allred score (hazard ratio 0.80, 95% CI 0.73–0.89).

**Conclusion:**

The histologic tumor type, ductal or lobular, determines DFS in hormone receptor-positive, HER2-negative, pT1-3 breast cancer besides the ER Allred score, LVSI and pN3.

## Introduction

Breast cancer is a heterogenous disease, morphologically and genomically, implying clinical consequences. Invasive ductal cancer (IDC, recently by the WHO reclassified as invasive carcinoma NST) is histopathologically the most frequent breast cancer, followed by invasive lobular cancer (ILC). Most of these tumors are hormone receptor-positive (HR +) and human epidermal growth receptor 2-negative (HER2-). In daily clinical practice this type of tumors represents the vast majority. Their treatment is based on clinical studies, but in general no difference is made between both types of invasive cancers. Nevertheless, they might behave differently. Lobular cancers are characterized by a lack of E-cadherin expression [1, 2] in combination with a high percentage of estrogen receptor (ER)-positivity [3–6]. They tend to have a more infiltrative growth, indistinct borders and are often detected as a larger tumor [6–8]. In accordance with the high positivity for the ER there is a marked sensitivity for endocrine therapy [9]. A recent study found chemotherapy to be less effective [10], and different survival rates are described [4, 11–15].

We examined whether the histologic tumor type, IDC or ILC, besides routine clinical and histopathological findings contributed to differences in outcomes of HR + , HER2- breast cancer without distant metastasis. Together with pT1-pT2 cancers, tumors with a pT3 classification were included, as ILC is often a large tumor at detection.

## Material and methods

Clinical and histopathological data of breast cancer patients treated in the Ghent University Hospital for the period 2001–2015 were recorded retrospectively in a database using ICD-10 coding and the TNM staging system. The raw dataset was verified by two independent researchers (MG and GB). The study was approved by the Ethical Commission of the Ghent University Hospital (reference number EC/2017/0287).

Following diagnosis by punction, staging and subsequent surgery, histopathological exams were performed and reported in a standardized manner.

Immunohistochemical staining for ER, progesterone receptor (PR) and HER2 just as the fluorescent in situ hybridization (FISH) for HER2 have been described previously [16]. The Allred score was assessed as described [17, 18] and considered positive as ≥ 1% of the cancer cells stained. In case of an immunoscore of 2 + or 3 + for HER2, the samples were subjected to a FISH procedure and those with amplification were considered as HER2 + . Histological diagnosis of ILC by morphologic appearance combined with loss or aberrant staining for E-cadherin and cytoplasmic localization of p120-catenin was validated as part of a multicentric study with 27 European institutions [19].

In total 3044 patients were diagnosed with breast cancer without metastasis. Following exclusion of pT4-tumors and restriction of the histological tumor type, IDC or ILC, 2429 patients were retained. Testing for HER2 was positive in 217 of 2141 patients with IDC and 6 of 288 ILC patients. Further 318 patients were HR-negative and 142 were lost to follow-up. The remaining collective of tumors were HR + , HER2-, stage pT1-3 and without distant metastasis, and consisted of 1586 patients with IDC and 257 with ILC. Subsequently, all files were analyzed in SPSS, version 26. Group comparison was performed using χ2 testing, except when less than 5 observations were in a cell, then a Fisher’s exact test was performed. Disease-free survival (DFS) was the time period in months from diagnosis until the occurrence of contralateral manifestation, recurrence, distant metastasis or death without previous symptoms. Overall-survival (OS) was the time between diagnosis and death due to any cause. Both were limited to 120 months. Kaplan–Meier curves were reported and differences in survival calculated using the log-rank test. Using Cox regression analysis uni- and multivariable models were calculated for DFS and OS [20] and reported as hazard ratio with 95% confidence intervals (95% CI) and corresponding p-value.

## Results

In total 1843 patients with a HR + , HER2-, pT1-3 IDC or ILC tumor without distant metastasis were identified and all cases were analyzed, as shown in Table 1. IDC represented 86.1% of these cases and the remaining 13.9% were ILC. Age distribution was similar for both subgroups. Most IDC tumors were detected in pT1 stage (69.9%), while ILC was mostly larger with 58.7% in stage pT2 or pT3. The pN0 stage was found in 64.2% of the IDC cases, whereas this was less for ILC (57.4%). LVSI proved to be more common in IDC with 34.6% vs. 25.5% for ILC. In our collective most ILCs were grade 2 (73.9%), and IDC had more grade 1 and 3 tumors. A high ER Allred score (7 or 8) was present in 94.5% of ILC, which was significantly more than for IDC (89.7%). Due to semi-recent introduction of Ki-67 many missing values were noted. ILC tumors had more often a Ki-67 under 20% than their IDC counterparts, 82.0% and 60.4% respectively.

**Table 1:** Patient characteristics with HR + , HER2-, pT1-3 invasive ductal and lobular cancer

	IDC	ILC	*p*-value*
	n (%)	n (%)	
Patients	1586 (86.1 of total)	257 (13.9 of total)	
*Age (years)*			0.26
≤ 39	88 (5.5)	7 (2.7)	
40–49	334 (21.1)	57 (22.2)	
50–59	476 (30.0)	70 (27.2)	
60–69	403 (25.4)	70 (27.2)	
≥ 70	285 (18.0)	53 (20.6)	
*pT*			< 0.001
1	1109 (69.9)	106 (41.2)	
2	439 (27.7)	118 (45.9)	
3	38 (2.4)	33 (12.8)	
*pN*			0.02
0	988 (64.2)	144 (57.4)	
1	418 (27.2)	71 (28.3)	
2	99 (6.4)	25 (10.0)	
3	34 (2.2)	11 (4.4)	
Missing	47	6	
*LVSI*			0.009
No	878 (65.4)	155 (74.5)	
Yes	465 (34.6)	53 (25.5)	
Missing	243	49	
*Grading*			< 0.001
1	220 (14.2)	8 (3.8)	
2	828 (53.6)	156 (73.9)	
3	498 (32.2)	47 (22.3)	
Missing	40	46	
*ER Allred score*			0.028
≤ 6	104 (10.3)	12 (5.5)	
7–8	906 (89.7)	206 (94.5)	
Missing	576	29	
*Ki-67 (%)*			< 0.001
≤ 9	124 (27.7)	42 (37.8)	
10–19	146 (32.7)	49 (44.1)	
≥ 20	177 (39.6)	20 (18.0)	
Missing	1139	146	

Percentages are calculated within each group, i.e. IDC or ILC, except for the number of “Patients” indicated as % of total

*IDC* invasive ductal cancer, *ILC* invasive lobular cancer, *pT* pathological tumor size, *pN* pathological lymph node status, *LVSI* lymphovascular space invasion, *ER* estrogen receptor

^*^p-value for χ2 test

In Table 2 the various types of treatment are summarized. Nearly all patients had surgery. Somewhat surprisingly, the percentage of patients receiving adjuvant chemotherapy was higher for ILC (42.8%) than for IDC (35.2%). Radiotherapy was more frequently applied in IDC than ILC (79.5% vs. 72.9%, respectively). Within the line of expectations as HR + was a prerequisite, nearly all patients were submitted to endocrine therapy.

**Table 2:** Treatment of patients with HR + , HER2-, pT1-3 invasive ductal or lobular cancer

	IDC	ILC	*p*-value*
	n (%)	n (%)	
*Surgery*			0.52
No	4 (0.3)	1 (0.4)	
Yes	1581 (99.7)	256 (99.6)	
Missing	1	0	
*Adjuvant chemotherapy*			0.022
No	1024 (64.8)	147 (57.2)	
Yes	556 (35.2)	110 (42.8)	
Missing	6	0	
*Radiotherapy*			0.018
No	321 (20.5)	68 (27.1)	
Yes	1247 (79.5)	183 (72.9)	
Missing	18	6	
*Endocrine therapy*			0.1
No	81 (5.1)	7 (2.8)	
Yes	1497 (94.9)	247 (97.2)	
Missing	8	3	

Percentages are calculated within each group, ie. IDC or ILC

*HR* hormone receptor, *HER2* human epithelial growth factor receptor 2, *IDC* invasive ductal cancer, *ILC* invasive lobular cancer, *pT* pathological tumor size

^*^p-value for χ^2^ test, except when less than 5 observed in any cell then Fisher’s exact test

**Fig. 1:**
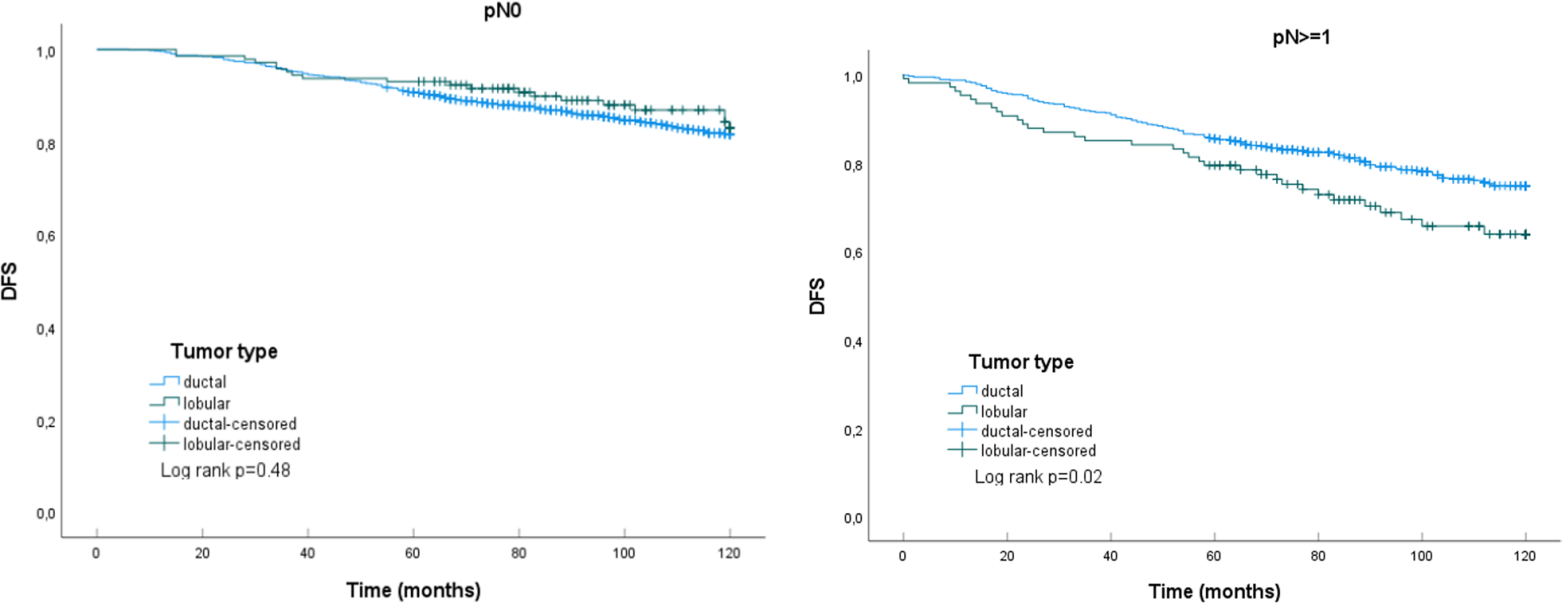
Kaplan–Meier curves for DFS of patients with HR + , HER2-, pT1-3 IDC or ILC. DFS as fraction of total number of patients with IDC (n = 984) or ILC (n = 144) and pN0, log rank test not significant (left) and of patients with IDC (n = 551) or ILC (n = 107) and pN ≥ 1, log rank test p = 0.02 (right)

**Fig. 2:**
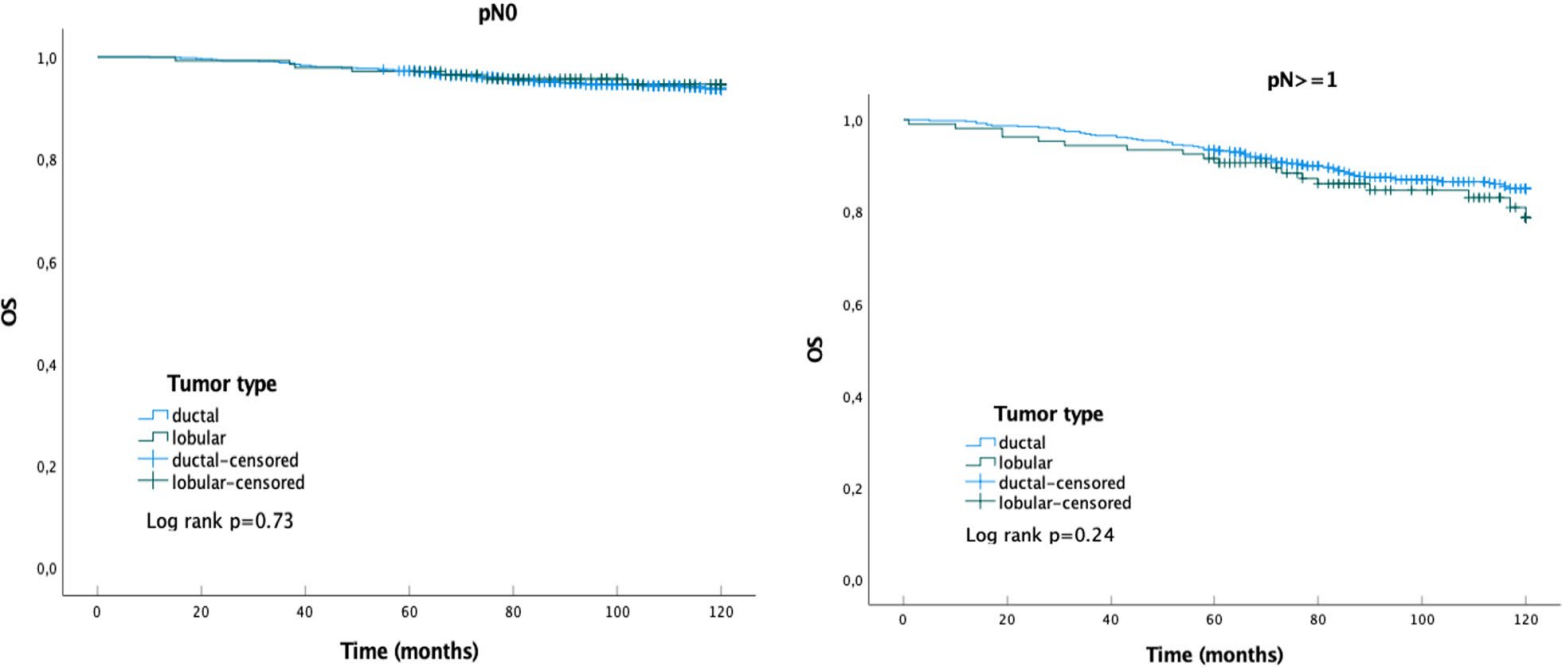
Kaplan–Meier curves for OS of patients with HR + , HER2-, pT1-3 IDC or ILC. OS as fraction of total number of patients with IDC (n = 984) or ILC (n = 144) and pN0, log rank test not significant (left) and of patients with IDC (n = 551) or ILC (n = 107) and pN ≥ 1, log rank test not significant (right)

Figure 1 shows the Kaplan–Meier curves for the histologic tumor type, lobular or ductal, and DFS. In case of pN0, the DFS for ILC was better than for IDC although not significant. A positive lymph node status (pN ≥ 1) resulted, however, for ILC in a worse DFS than for IDC (*p* = 0.02). No differences in Kaplan–Meier curves for the OS and histologic tumor type, ILC or IDC, could be observed (Fig. 2).

Table 3 represents the models for DFS and OS using uni- and multivariable Cox regression analysis. In the univariable analysis the majority of clinical and histopathological variables were significant determinants of DFS and OS. In the subsequent multivariable analysis only a few of them remained significant. The multivariable analysis for DFS showed the histologic tumor type, IDC or ILC, to be an explanatory variable. The tumor type ILC had a 77% higher risk for an event (*p* = 0.02) than IDC. Next was pN3 associated with a hazard risk of 2.29 compared to pN0 (*p* = 0.04). The presence of LVSI increased the risk of an event (hazard ratio 1.75, *p* = 0.01), whereas the Allred score decreased it (hazard ratio 0.84, *p* < 0.001). Other variables, such as tumor size, grading and Ki-67 were not contributive. In the multivariable analysis for OS the variables age (hazard ratio 1.05, *p* < 0.001) next to LVSI (hazard ratio 3.62, *p* < 0.001) and the Allred score (hazard ratio 0.80, *p* < 0.001) were explanatory risk factors. The histologic tumor type and other variables like tumor size were no determinants.

**Table 3:** Uni- and multivariable Cox regression analysis for DFS and OS for patients with HR + , HER2-, pT1-3 IDC or ILC

Variable	Univariable analysis			Multivariable analysis		
	Hazard ratio (95% CI)		p-value	Hazard ratio (95% CI)		p-value
Model for disease-free survival (120 months)						
Age (years)	0.99	(0.99–1.00)	0.20			
*pT*			< 0.001			
1 (reference)	1.00					
2	1.59	(1.28–1.97)	< 0.001			
3	1.66	(1.01–2.72)	0.04			
*pN*			< 0.001			0.20
0 (reference)	1.00			1.00		
1	1.30	(1.02_1.65)	0.03	1.20	(0.73–1.97)	0.46
2	2.25	(1.60–3.18)	< 0.001	1.50	(0.74–3.02)	0.26
3	4.61	(2.96–7.18)	< 0.001	2.29	(1.03–5.09)	0.04
*Histologic tumor type*						
Ductal (reference)	1.00			1.00		
Lobular	1.19	(0.90–1.59)	0.20	1.77	(1.08–2.90)	0.02
*Grading*			0.004			
1 (reference)	1.00					
2	1.29	(0.88–1.89)	0.19			
3	1.76	(1.19–2.60)	0.005			
*LVSI*						
No (reference)	1.00			1.00		
Yes	1.78	(1.39–2.28)	< 0.001	1.75	(1.12–2.74)	0.01
Ki-67	1.01	(1.00–1.02)	0.02			
ER Allred score	0.83	(0.78–0.89)	< 0.001	0.84	(0.78–0.91)	< 0.001
PR Allred score	0.94	(0.89–1.00)	0.07			
*Model for overall survival (120 months)*						
Age (years)	1.03	(1.01–1.04)	< 0.001	1.05	(1.02–1.07)	< 0.001
pT			< 0.001			
1 (reference)	1.00					
2	2.16	(1.58–2.95)	< 0.001			
3	1.41	(0.62–3.25)	0.49			
*pN*			< 0.001			
0 (reference)	1.00					
1	1.96	(1.36–2.82)	< 0.001			
2	4.35	(2.77–6.83)	< 0.001			
3	7.42	(4.16–13.25)	< 0.001			
Histologic tumor type						
Ductal (reference)	1.00					
Lobular	1.24	(0.81–1.88)	0.32			
Grading			0.014			
1 (reference)	1.00					
2	1.68	(0.90–3.16)	0.11			
3	2.36	(1.24–4.49)	0.009			
*LVSI*						
No (reference)	1.00			1.00		
Yes	2.34	(1.64–3.34)	< 0.001	3.62	(1.92–6.82)	< 0.001
Ki-67	1.02	(1.00–1.04)	0.01			
ER Allred score	0.81	(0.74–0.88)	< 0.001	0.80	(0.73–0.89)	< 0.001
PR Allred score	0.94	(0.85–1.03)	0.19			

__________________________________________________________________________________________

*HR* hormone receptor, *HER2* human growth factor receptor 2, *CI* confidence interval, *pT* pathological tumor status, *pN* pathological lymph node status, *LVSI* lymphovascular space invasion, *ER* estrogen receptor, *PR* progesteron receptor

## Discussion

This study on patients with HR + , HER2-, pT1-3 breast cancer shows the histologic invasive tumor type, ductal or lobular, to be a determinant for DFS over 10 years in a multivariable Cox regression analysis. LVSI and the ER Allred score were determinants for both DFS and OS over ten years, whereas pN3 was only a determinant for DFS and age only for OS. In addition, Kaplan–Meier curves for DFS showed a worse outcome for the histologic tumor type ILC with lymph node metastasis.

The percentage of patients with ILC (13.9%) is in accordance with the findings of other studies [21, 22]. Patients with ILC had larger tumors and more often a positive axillary lymph node status than those with IDC. Previous studies found similar results [3, 13, 23, 24] and can be explained by their infiltrative character resulting in difficult diagnosis on palpation and by mammography [7]. Furthermore, the large tumor size and positive lymph node status might explain the somewhat higher percentage of administered chemotherapy for ILC in our collective.

In addition, this study found high ER Allred scores in the great majority of ILC and less for IDC, and hence, helps to explain the good sensitivity of ILC towards endocrine therapy [9]. The high percentage of ER positivity for ILC is a constant finding over several studies [3–6, 9]. The recent study of Zhao [25] on the SEER database 2004–2015 with 144,651 IDC and 16,433 ILC reported HR positivity (including HER2-positive tumors) in 82.7% of the IDC and 98.6% of the ILC patients. The percentages for HR + /HER2-negative IDC and ILC tumors were 71.3 and 94.5, respectively.

The majority of the lobular tumors had a low Ki-67 (82%) compared to their ductal counterparts (60.4%) which has been documented before [26, 27]. Due to the introduction of Ki-67 in recent years, older cases had no Ki-67 staining which explains the high number of missing values. These missing values bring about the corresponding cases to be omitted from the Cox regression analysis. In the univariable analysis Ki-67 had a hazard ratio of only 1.01 (95% CI 1.00–1.02, *p* = 0.02) and was not of significance in the multivariable model. Obviously, Ki-67 was not found to be a determinant of DFS or OS in this confined collective.

According to Table 2 nearly all patients, independent of the histologic tumor type, had surgery. In our study the type of surgery, breast conserving or mastectomy, was not specified.

Similarly, nearly all patients had endocrine therapy. In 2001–2015, the administration of tamoxifen or an aromatase-inhibitor for five years was standard. But nowadays, the strict therapeutic landscape has evolved. Ovarian suppression can be offered to premenopausal patients at risk. Extended endocrine therapy for lLC or IDC with risk factors is a frequently used option [28]. Furthermore, gene expression profiles for assessing the benefit of adjuvant chemotherapy have become widely available. Nevertheless, the major advantage of the strict set-up of the endocrine therapy back at that time allows for a good comparability between groups, such as for the tumor type, in this study.

Radiotherapy was less frequent in ILC than IDC, respectively 72.9% vs. 79.5%, and is probably related to the type of surgery. In ILC, more mastectomies are described [3, 23, 29–31], omitting the need for breast radiation.

Different results about survival in ILC compared to IDC have been described without a corresponding adequate explanation [4, 11–15, 25]. In our study the inclusion criteria were narrowed down to HR + , HER2- pT1-3 IDC or ILC. This collective represents the bulk of tumors in clinical practice. As described earlier, Zhao [25] found 71.3% of IDC and 94.5% of ILC in the SEER database to be HR + /HER2-. By omitting triple negative as well as HER2 + breast cancer the survival curves will not be governed anymore by outliers with an unfavorable prognosis. In our chosen collective of HR + /HER2- tumors the survival curves of IDC will have ameliorated more than those of ILC as IDC contained in Zhao’s publication 28.7% triple negative and HER2 + tumors whereas this was for ILC just 5.5%.

Using exactly these criteria the subsequent Kaplan–Meier curves showed in case of a positive lymph node status the DFS, but not OS, of the histologic tumor type ILC to be significant worse than for IDC. In case of pN0 the DFS or OS for both tumor types did not differ significantly. Adachi et al. [14] studied luminal tumors defined as ER + and HER2- and found similar results with a worse DFS and OS for node-positive ILC compared to IDC. In this study the node-positive ILC group demonstrated even a worsening of the DFS after 60 months. Obviously, in this group an excess of high-risk ILCs were present. In addition, stopping of the endocrine therapy after 5 years at that time in 2001–2015 might also help to explain this observation. Nowadays endocrine therapy for ILC is at least five years, preferably 7–10 years according to international guidelines [28].

Next step in the survival analysis showed nearly all clinical and histopathological parameters in the univariable analysis to be significant. Other publications have similar conclusions, including an increased risk for ILC in luminal tumors, defined as HR + , HER2- [14, 26].

By including the ER Allred score in combination with the histologic tumor type in the multivariable Cox regression analysis for DFS over 10 years, a significant difference for the histologic tumor type, ILC or IDC, could be determined. With IDC as reference, the tumor type ILC gave an additional risk of 77% for an event. On the other hand for each arbitrary unit of the ER Allred score there was a risk reduction of 16%. Most of the ILCs had high ER Allred scores, whereas this was not the case for IDC. In case of ILC with the maximum Allred score, the risk reduction was substantial. For this reason most ILCs had a very good DFS, although the histologic tumor type ILC itself was a risk factor. Other studies applying a multivariable analysis on their data were either in a different patient population or did not take the ER Allred score into account [14, 26, 27]. Interestingly, Adachi et al. [14] found a significant increased risk for the tumor type ILC in the multivariable analysis following the inclusion of endocrine therapy and chemotherapy, which however should be regarded as confounders. Flores-Diaz et al. [27] described an increased hazard ratio (1.6, *p* = 0.017) for the tumor type ILC, but also included the phenotype (hormone-sensitive, triple negative or HER2 +) in the multivariable DFS analysis. In our study LVSI and pN3 were other determinants of DFS over 10 years. Not identifying the grade as a determinant might be surprising. The prognosis of ILC is considered to be good as ILCs are likely to be low grade [3, 4, 9, 11, 25, 26, 32]. In our study, most ILCs were classified as grade 2. Metzger-Fihlo et al. [33] reassessed the histological grade (HG) of 166 ILC samples using the Genomic Grade gene expression profile (GG). The HG classification for grade 1, 2 and 3 was 20%, 73% and 7%, respectively. Using the Genomic Grade, the problematic group of G2 was reduced: 64% for GG1, 19% for GG2 and 17% for GG3. In a multivariable Cox proportional hazards model, GG2/GG3 proved to be a significant prognostic factor for DFS and OS. Histological assessment of the grade in ILC seems to be difficult and compromises the value of grading as a prognostic factor.

For the OS over 10 years LVSI, the ER Allred score, and age played a major role but not the histologic tumor type. These findings are not extraordinary when considering studies about aromatase-inhibitors. These studies show significant results for the effect of aromatase-inhibitors on DFS, but continuation of these studies to evaluate the effect of aromatase-inhibitors on OS did not reveal significant results as other death causes started to prevail.

In a recent publication Zhao [25] analyzed 171,881 patients with IDC, ILC and mixed IDC and ILC (IDLC) in the SEER database. In a Cox regression analysis, the tumor types ILC and IDLC were determinants for OS (hazard ratio 0.84, 95% CI 0.77–0.90 and hazard ratio 0.91, 95% CI 0.83–1.00) although this was not the case for breast cancer specific survival. In Zhao’s Cox model there were no restrictions for the tumors examined: triple negative, HER2 positive and T4 tumors were included. Further, no ER Allred score was utilized next to the tumor type, just ER-positivity, which was highly significant. These divergences led inevitably to differences in the observed findings. Another factor of interest is the not specified number of months for OS in the Cox model by Zhao, but might be 5 years according to the Kaplan–Meier curves. We observed, however, a further worsening of DFS for node-positive ILC 60 months after initial diagnosis. By reducing the survival time to 5 years the subsequent multivariable analysis by Zhao will have resulted in a reduced risk for ILC and is another factors explaining differences in the results. In the study by Timbres et al. [34] the follow-up was till 20 years and the OS of patients with ER + IDC and ILC was analyzed. T4 tumors were, however, included. One of the Cox proportional hazard analyses comprised 784 patients with ER + HER2- IDC or ILC following adjuvant or neoadjuvant chemotherapy. ILC had an increased hazard ratio of 1.46 (95% CI 1.06–1.93) vs. IDC.

Invasive lobular cancer is different from ductal cancer in clinical appearance, imaging, histopathological findings, treatment options and survival. This histologic tumor type needs clarification in many ways. The underlying causes of its high hormone sensitivity and chemoresistance are challenges to explore. In this study data were well documented and collected carefully allowing a substantial analysis between groups. The study itself is retrospective and single-institutional, but the risk of bias is low, as the medical setup and the reporting of routine clinical features and histopathological findings was standardized and uniform. Furthermore, therapeutic schemes were rather straightforward at that time. By restriction to HR + , HER2-, pT1-3, ductal and lobular cancers without distant metastasis, tumors with an unfavorable prognosis, such as triple negative and HER2 + breast cancer, were omitted especially in the IDC group. ILC tumors had different characteristics than IDC: larger in size, more grade 2 tumors, low Ki-67 and high ER Allred score. Using Kaplan–Meier curves lymph node-positive ILC showed a worse DFS than the corresponding IDC. In the multivariable analysis the tumor type, ductal or lobular, was proven to be a determinant of DFS just as LVSI, the ER Allred score for DFS and OS, pN3 for DFS and age for OS. Face it, in terms of survival ILC and IDC are different, next to other determinants related to the potential to metastasize, hormone receptor-status and age.

## Data Availability

The data that support the findings of this study are available from professor Geert Braems, but restrictions apply to the availability of these data, which were used under license for the current study, and so are not publicly available. Data are however available from the authors upon reasonable request and with permission of prof. Geert Braems.

## Abbreviations

IDC: Invasive ductal cancer
ILC: Invasive lobular carcinoma
HR +: Hormone receptor-positive
HER2: Human epidermal growth receptor 2-negative
ER: Estrogen receptor
PR: Progesterone receptor
LVSI: Lymphovascular space invasion
DFS: Disease free survival
OS: Overall survival
pT: Pathological tumor size
pN: Pathological lymph node status
CI: Confidence interval

## References

[1] De Leeuw WJF, Berx G, Vos CBJ, Peterse JL, Van De Vijver MJ, Litvinov S (1997). Simultaneous loss of E-cadherin and catenins in invasive lobular breast cancer and lobular carcinoma in situ. J Pathol.

[2] Reed AEM, Kutasovic JR, Lakhani SR, Simpson PT (2015). Invasive lobular carcinoma of the breast: morphology, biomarkers and ’omics. Breast Cancer Res.

[3] Arpino G, Bardou VJ, Clark GM, Elledge RM (2004). Infiltrating lobular carcinoma of the breast: tumor characteristics and clinical outcome. Breast Cancer Res.

[4] Wasif N, Maggard MA, Ko CY, Giuliano AE (2010). Invasive lobular vs. ductal breast cancer: a stage-matched comparison of outcomes. Ann Surg Oncol.

[5] Wang K, Zhu GQ, Shi Y, Li ZY, Zhang X, Li HY (2019). Long-term survival differences between T1–2 invasive lobular breast cancer and corresponding ductal carcinoma after breast-conserving surgery: a propensity-scored matched longitudinal cohort study. Clin Breast Cancer.

[6] Yang C, Lei C, Zhang Y, Zhang J, Ji F, Pan W (2020). Comparison of overall survival between invasive lobular breast carcinoma and invasive ductal breast carcinoma: a propensity score matching study based on SEER. Database Front Oncol.

[7] Johnson K, Sarma D, Hwang ES (2015). Lobular breast cancer series: Imaging. Breast Cancer Res.

[8] Mann RM, Hoogeveen YL, Blickman JG, Boetes C (2008). MRI compared to conventional diagnostic work-up in the detection and evaluation of invasive lobular carcinoma of the breast: a review of existing literature. Breast Cancer Res Treat.

[9] Rakha EA, El-Sayed ME, Powe DG, Green AR, Habashy H, Grainge MJ (2008). Invasive lobular carcinoma of the breast: response to hormonal therapy and outcomes. Eur J Cancer.

[10] Marmor S, Hui JYC, Huang JL, Kizy S, Beckwith H, Blaes AH (2017). Relative effectiveness of adjuvant chemotherapy for invasive lobular compared with invasive ductal carcinoma of the breast. Cancer.

[11] García-Fernández A, Lain JM, Chabrera C, García Font M, Fraile M, Barco I (2015). Comparative long-term study of a large series of patients with invasive ductal carcinoma and invasive lobular carcinoma. Loco-regional recurrence, metastasis, and survival. Breast J.

[12] Biglia N, Maggiorotto F, Liberale V, Bounous VE, Sgro LG, Pecchio S (2013). Clinical-pathologic features, long term-outcome and surgical treatment in a large series of patients with invasive lobular carcinoma (ILC) and invasive ductal carcinoma (IDC). Eur J Surg Oncol.

[13] Molland JG, Donnellan M, Janu NC, Carmalt HL, Kennedy CW, Gillett DJ (2004). Infiltrating lobular carcinomaFa comparison of diagnosis, management and outcome with infiltrating duct carcinoma. The Breast.

[14] Adachi Y, Ishiguro J, Kotani H, Hisada T, Ichikawa M, Gondo N (2016). Comparison of clinical outcomes between luminal invasive ductal carcinoma and luminal invasive lobular carcinoma. BMC Cancer.

[15] Mate TP, Carter D, Fischer DB, Hartman PV, McKhann C, Merino M (1986). A clinical and histopathologic analysis of the results of conservation surgery and radiation therapy in stage I and II breast carcinoma. Cancer.

[16] Lambein K, Praet M, Forsyth R, Van Den Broecke R, Braems G, Matthys B (2011). Relationship between pathological features, HER2 protein expression and HER2 and CEP17 copy number in breast cancer: Biological and methodological considerations. J Clin Pathol.

[17] Allred D, Clark G (1993). Association of p53 protein expression with tumor cell proliferation rate and clinical outcome in node-negative breast cancer. J Nat Cancer Inst.

[18] Hanby AM, Walker C. Tavassoli FA, Devilee P: Pathology and Genetics: Tumours of the Breast and Female Genital Organs. WHO Classification of Tumours series - volume IV. Lyon, France: IARC Press: 2003. 250pp. ISBN 92 832 2412 4. Breast Cancer Res 2004;6

[19] Christgen M, Kandt LD, Antonopoulos W, Bartels S, Van Bockstal MR, Bredt M (2022). Inter-observer agreement for the histological diagnosis of invasive lobular breast carcinoma. J Pathol Clin Res.

[20] Katz M. Multivariable analysis: a practical guide for clinicians and public health researchers 2011.

[21] Li CI, Anderson BO, Daling JR, Moe RE (2003). Trends in incidence rates of invasive lobular and ductal breast carcinoma. JAMA.

[22] Li CI, Daling JR (2007). Changes in breast cancer incidence rates in the United States by histologic subtype and race/ethnicity, 1995 to 2004. Cancer Epidemiol Biomarkers Prev.

[23] Pestalozzi BC, Zahrieh D, Mallon E, Gusterson BA, Price KN, Gelber RD (2008). Distinct clinical and prognostic features of infiltrating lobular carcinoma of the breast: combined results of 15 International Breast Cancer Study Group clinical trials. J Clin Oncol.

[24] Silverstein MJ, Lewinsky BS, Waisman JR, Gierson ED, Colburn WJ, Senofsky GM (1994). Infiltrating lobular carcinoma. Is it different from infiltrating duct carcinoma?. Cancer.

[25] Zhao H (2021). The prognosis of invasive ductal carcinoma, lobular carcinoma and mixed ductal and lobular carcinoma according to molecular subtypes of the breast. Breast Cancer.

[26] Corona SP, Bortul M, Scomersi S, Bigal C, Bottin C, Zanconati F (2020). Management of the axilla in breast cancer: outcome analysis in a series of ductal versus lobular invasive cancers. Breast Cancer Res Treat.

[27] Flores-Díaz D, Arce C, Flores-Luna L, Reynoso-Noveron N, Lara-Medina F, Matus JA (2019). Impact of invasive lobular carcinoma on long-term outcomes in Mexican breast cancer patients. Breast Cancer Res Treat.

[28] Burstein HJ, Lacchetti C, Anderson H, Buchholz TA, Davidson NE, Gelmon KA (2018). Adjuvant endocrine therapy for women with hormone receptor-positive breast cancer: ASCO clinical practice guideline focused update. J Clin Oncol.

[29] Yeatman TJ, Lyman GH, Smith SK, Reintgen DS, Cantor AB, Cox CE (1996). Bilaterality and recurrence rates for lobular breast cancer: considerations for treatment. Ann Surg Oncol.

[30] Zengel B, Yararbas U, Duran A, Uslu A, Elıyatkın N, Demırkıran MA, Cengiz F, Şimşek C, Postacı H, Vardar E, Durusoy R (2015). Comparison of the clinicopathological features of invasive ductal, invasive lobular, and mixed (invasive ductal + invasive lobular) carcinoma of the breast. Breast Cancer.

[31] Winchester DJ, Chang HR, Graves TA, Menck HR, Bland KI, Winchester DP (1998). A comparative analysis of lobular and ductal carcinoma of the breast: presentation, treatment, and outcomes. J Am Coll Surg.

[32] Toikkanen S, Pylkkänen L, Joensuu H (1997). Invasive lobular carcinoma of the breast has better short- and long-term survival than invasive ductal carcinoma. Br J Cancer.

[33] Metzger-Filho O, Michiels S, Bertucci F, Catteau A, Salgado R, Galant C (2013). Genomic grade adds prognostic value in invasive lobular carcinoma †. Ann Oncol.

[34] Timbres J, Moss C, Mera A, Haire A, Gillett C, Van HM (2021). Survival outcomes in invasive lobular carcinoma compared to oestrogen receptor-positive invasive ductal carcinoma 2021. Cancers.

